# Short-term physiologic response of the green microalga *Picochlorum* sp. (BPE23) to supra-optimal temperature

**DOI:** 10.1038/s41598-022-06954-6

**Published:** 2022-02-28

**Authors:** Robin Barten, Michelle Kleisman, Giulia D’Ermo, Harm Nijveen, Rene H. Wijffels, Maria J. Barbosa

**Affiliations:** 1 Bioprocess Engineering and AlgaePARC, Wageningen University and Research, PO Box 16, 6700 AA Wageningen, The Netherlands; 2 Bioinformatics Group, Wageningen University and Research, PO Box 633, 6700 AP Wageningen, The Netherlands; 3 Biosciences and Aquaculture, Nord University, 8049 Bodø, Norway

**Keywords:** Biotechnology, Transcriptomics, Plant stress responses, Heat, Applied microbiology, Gas chromatography

## Abstract

Photobioreactors heat up significantly during the day due to irradiation by sunlight. High temperatures affect cell physiology negatively, causing reduced growth and productivity. To elucidate the microalgal response to stressful supra-optimal temperature, we studied the physiology of *Picochlorum* sp. (BPE23) after increasing the growth temperature from 30 °C to 42 °C, whereas 38 °C is its optimal growth temperature. Cell growth, cell composition and mRNA expression patterns were regularly analyzed for 120 h after increasing the temperature. The supra-optimal temperature caused cell cycle arrest for 8 h, with concomitant changes in metabolic activity. Accumulation of fatty acids was observed during this period to store unspent energy which was otherwise used for growth. In addition, the microalgae changed their pigment and fatty acid composition. For example, palmitic acid (C16:0) content in the polar fatty acid fraction increased by 30%, hypothetically to reduce membrane fluidity to counteract the effect of increased temperature. After the relief of cell cycle arrest, the metabolic activity of *Picochlorum* sp. (BPE23) reduced significantly over time. A strong response in gene expression was observed directly after the increase in temperature, which was dampened in the remainder of the experiment. mRNA expression levels associated with pathways associated with genes acting in photosynthesis, carbon fixation, ribosome, citrate cycle, and biosynthesis of metabolites and amino acids were downregulated, whereas the proteasome, autophagy and endocytosis were upregulated.

## Introduction

Photobioreactors heat up significantly as a result of irradiation by sunlight, leading to shifts in temperature throughout the day up to levels that can be stressful for microalgae. Heat stress poses a significant challenge in the cultivation of microalgae for the production of food, feed and chemicals. Non-optimal temperatures reduce microalgal growth, thereby reducing productivity and photosynthetic efficiency^1^. The effect of excessive heat on the cellular physiology has been studied in photosynthetic model organisms such as *Arabidopsis thaliana* and *Chlamydomonas reinhardtii*. While many aspects of the response to heat have been studied, much still has to be learned on how exactly microalgae cope with heat^2–4^. In addition, detailed research on the effect of heat stress on other photosynthetic (micro)organisms, is scarce.

Many cellular processes are impacted by supra-optimal temperature which ultimately unbalances cellular homeostasis^5^. Several effects are; failure of correct protein folding and protein complex assembly, destabilization of cell membranes through increased membrane fluidity, a reduction in photosynthesis, changes in DNA replication and repair, changes in enzymatic activity, and reduced metabolic activity^2,6,7^. As a response to temperature-induced physiological changes, heat shock factors (HSF’s) and heat shock proteins (HSP’s) are expressed to mediate and protect against adverse effects through chaperoning after exposure to heat stress^4,5,8^. Excessive heat kills microalgae quickly due to denaturation of proteins and destabilization of membranes. However, in this paper we will focus on a supra-optimal temperature. Supra optimal temperatures fall between the optimal and maximal temperature for growth. Here, growth rate is reduced, but cells still survive.

Photosynthesis, carbon fixation and the central energy metabolism form a delicate system with consecutive enzymatic reactions^3,9^. Photosystem II is considered the most thermosensitive complex within the photosynthetic pathway while several components of the carbon fixation pathway are thermosensitive^2^. However, Photosystem II is not considered a bottleneck between the optimal and lethal growth temperature^9^. When one or more reactions in the energy metabolism are inhibited by temperature, the entire energy flux through the pathway will slow down. This ultimately results in excessive energy in the photosystems, which will overflow and lead to the formation of harmful reactive oxygen species (ROS)^3^. To prevent the accumulation of ROS, cells suppress photosynthesis by downregulation of key genes. Next to suppression of photosynthesis to prevent ROS formation, mechanisms for ROS scavenging are activated. ROS scavenging in microalgae is done through several mechanisms: enzymatically (by catalases, superoxide dismutases, and peroxidases) and by production of antioxidants for ROS scavenging such as carotenoids^10,11^. A third protection mechanism, to prevent formation of ROS, is de novo production and accumulation of energy storage compounds such as lipids and carbohydrates as an energy sink^2,4^. As a result of the before mentioned impact of temperature and the response mechanisms, growth rate decreases^12,13^.

In this study, we investigated the effect of supra-optimal temperature on the physiology of *Picochlorum* sp. (BPE23) to elucidate how this microalga copes and acclimates to temperature stress. *Picochlorum* sp. (BPE23) exhibits a high maximal growth rate of 5 d^−1^ under non-limiting growth conditions which together with its robustness and its relatively small genome size of ~ 13 Mbp makes it an interesting model platform for both fundamental studies, and industrial application^14–17^. This chlorophyte has a cell size of 3–4 µm. Currently, five genome assemblies for various species of *Picochlorum* are available and tools for genetic modification have been developed^17^. A temperature increase from 30 to 42 °C was studied, which is 4 °C above the optimal growth temperature of this strain^14^. To elucidate the effect of the temperature shift to 42 °C, the growth rate, cell volume, quantum yield, pigment composition, fatty acid composition, and mRNA expression were periodically measured for 120 h. The results show how *Picochlorum* sp. (BPE23) copes with the immediate adverse effects of supra-optimal temperatures to prevent cell damage and how the onset to acclimatization to this temperature takes place.

## Results

### Temperature increase to supra-optimal level leads to cell-cycle arrest and decreased growth

**Figure 1:**
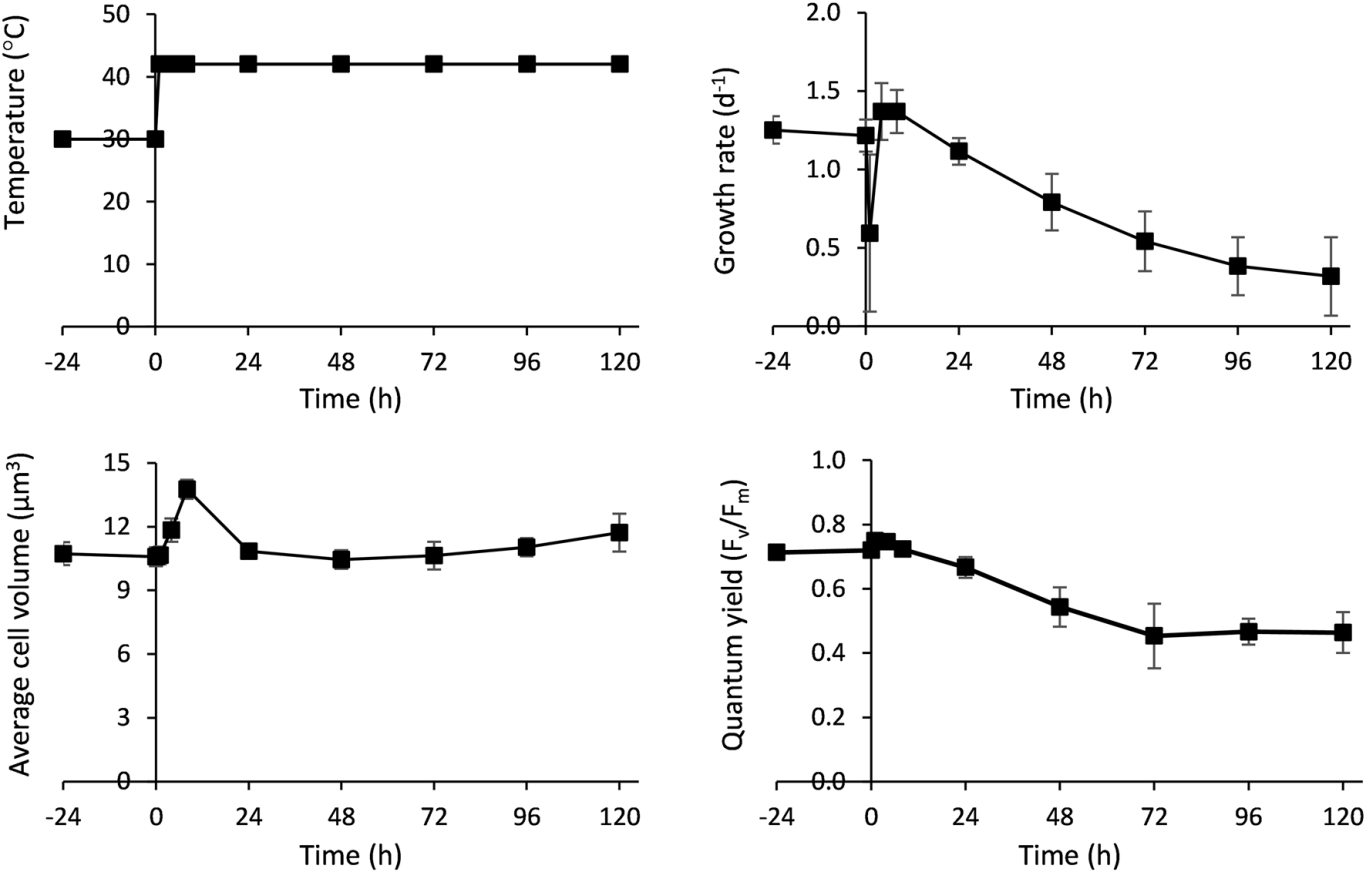
Effect of temperature increase to supra-optimal value, on the specific growth rate (d^−1^), quantum yield (F_v_/F_m_), and average cell volume (μm^3^ cell^−1^) in Picochlorum sp. (BPE23). Temperature was increased from steady state growth at 30–42 °C at Time = 0 h. Data represents the mean ± standard deviation of biological triplicate experiments.

The effect of the increase in temperature from 30 to 42 °C for a time span of 120 h on *Picochlorum* sp. (BPE23) can be seen in Fig. 1. The specific growth rate, quantum yield, and cell volume were measured over time, before and after temperature increase. At the onset of temperature increase (t = 0), the cell culture was at steady state, in a photobioreactor operated in turbidostat mode. Since only temperature was changed, it is safely assumed that the observed changes in the culture are solely due to the temperature shift. Two distinct responses were observed; an immediate short-term response, followed by a long-term response in which the microalgae gradually acclimated.

One hour after the temperature shift all monitored growth parameters had changed (Fig. 1). The specific growth rate decreased during the first hour, followed by a temporary increase to higher levels than those observed at 30 °C. It remained high until 8 h after the temperature shift point at which it started to decrease until the end of the experiment (120 h). During the first 8 h the average cell volume increased by 30%, from 10.6 to 13.76 µm^3^. The combination of a decreased growth rate and an increase in cell volume due to halted cell division indicates a cell cycle arrest. The cell cycle arrest was released after 8 h and cell volume returned to the initial value after 24 h. The cell volume gradually kept increasing from 24 h after the temperature shift until the end of the experiment.

Quantum yield (F_v_/F_m_) is a measure for the maximum photosynthetic capacity of photosystem II and can be used to monitor photo inhibitory damage in response to stress, in this study as a result of high temperature^18^. The observed quantum yield shortly increased from 0.72 to 0.75 at 42 °C, after which a gradual decrease was observed to a level of 0.45 at 72 h and remained stable until the end of the experiment (120 h).

### Pigment content increased after exposure to supra-optimal temperature

**Figure 2:**
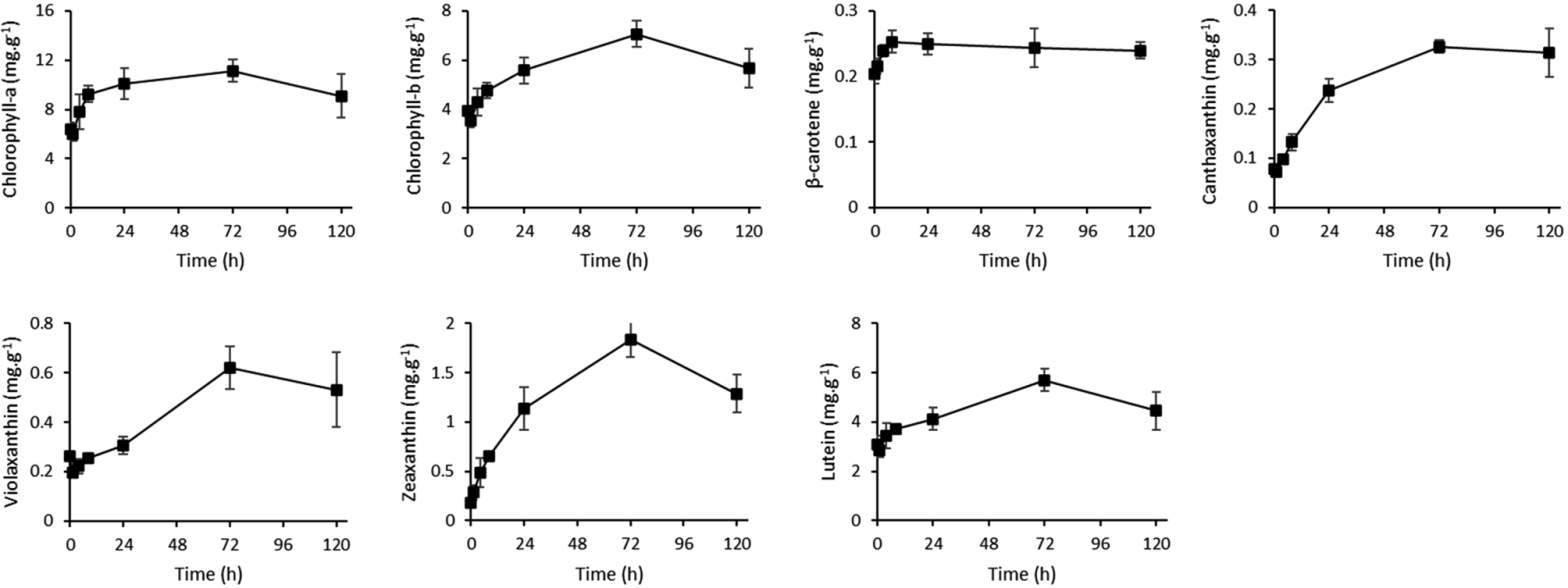
Concentrations of chlorophyll-a, chlorophyll-b, β-carotene, canthaxanthin, violaxanthin, zeaxanthin, and lutein in algal biomass as measured over time, starting at the moment of the temperature increase from 30 to 42 °C. Data represents the mean ± standard deviation of biological triplicate experiments.

Pigment content was measured over time and is displayed in Fig. 2. The pigment concentrations were also measured through optical density measurements which show a comparable trend (Supplementary material 5). The concentration of chlorophyll-a and chlorophyll-b decreased slightly in the first hour after the temperature shift. However, from 4 h onwards an increase in concentration was observed with a peak at 72 h. The concentration for both chlorophyll-a and chlorophyll-b increased with a factor of 1.75, indicating an upregulation of photosystems. The ratio between chlorophyll-a and chlorophyll-b increased slightly between 8 and 24 h, but returned to the values as found at the start of the experiment at 72 h.

Carotenoids, in addition to a light-harvesting function within the photosystems, also have a photoprotective role in non-photochemical quenching, ROS scavenging, and/or filtering of light^19^. The xanthophyll pigments Violaxanthin and Zeaxanthin show a peak concentration at 72 h after temperature increase. Antheraxanthin was measured but not detected. The concentration of zeaxanthin increased directly after exposure to supra-optimal temperature, from 0.18 to 1.84 mg g^−1^ after 72 h, whereas the concentration of violaxanthin initially decreases after 1 h, followed by a gradual increase during the first 24 h. Xanthophyll pigments accommodate energy dissipation through non-photochemical quenching^19^. Also, β-carotene, Canthaxanthin and Lutein, known as strong antioxidants, were found at increased concentrations in stressed cells after exposure to supra-optimal temperatures^19^.

### *Picochlorum* sp. (BPE23) accumulated fatty acids as a response to the increased temperature

**Figure 3:**
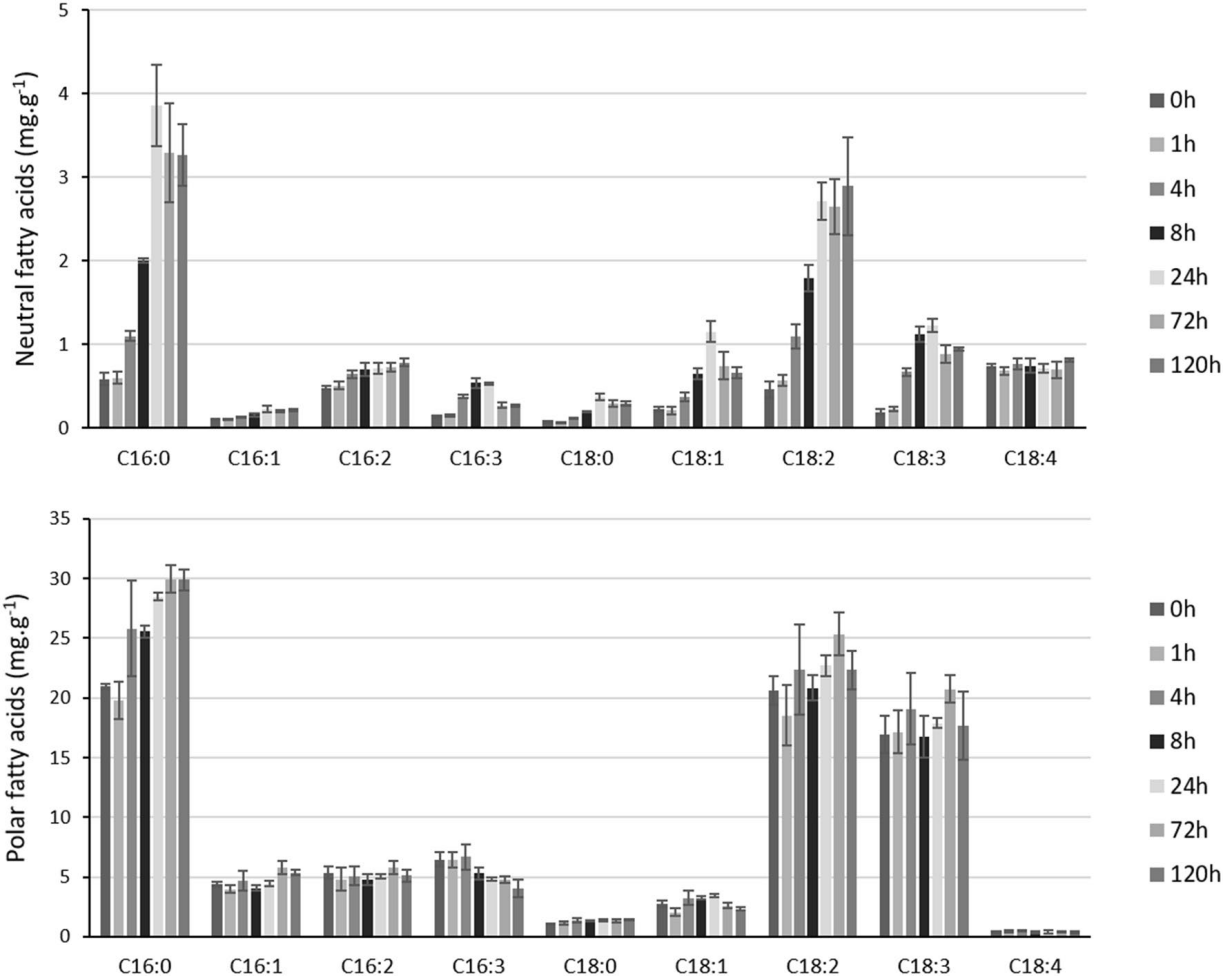
Neutral fatty acid and polar fatty acid concentrations as measured over time, starting at the moment of the temperature increase from 30 to 42 °C. Data represents the mean ± standard deviation of biological triplicate experiments.

Fatty acids can be distributed into two general groups; neutral and polar, which are present as lipid bodies and in the cell membranes, respectively (Fig. 3). The concentration of neutral fatty acids increased from 3.0 mg g^−1^ at 0 h to 11.5 mg g^−1^ at 24 h, whereas the total content of polar fatty acids increased from 79.0 to 88 mg g^−1^. Within the neutral fatty acids, all fatty acid species showed an increased concentration at approximately the same ratio. In the polar fatty acids the increase in content was mainly caused by C16:0 content, which C16:0 increased from 21.0 mg g^−1^ after 0 h to 30.0 mg g^−1^ after 72 h. At the same time, a decrease in C16:3 was observed over time.

### A large transcriptional change was observed after the increase in temperature

Comparative transcriptome analysis provided further insight into the stress response of *Picochlorum* sp. (BPE23) to supra-optimal temperatures. In total 6990 genes were identified in the transcriptome, of which 5930 were annotated through BLASTP and Orthofinder search and inferring domains. In addition, 55 novel genes were identified. 4427 of these genes matched to *A. thaliana* proteins (62.8%). Iteratively, the remaining unidentified genes were matched to other species of which 678 genes matched to *C. Variabilis* orthologs (9.6%), 116 genes matched to *A. protothecoides* orthologs (1.6%), 3 genes matched to *Helicosporidium* sp. orthologs (0.04%) and 60 genes matched to *C. reinhardtii* orthologs (0.9%). 643 out of the remaining 1761 genes had a domain annotation (9.1%).

**Figure 4:**
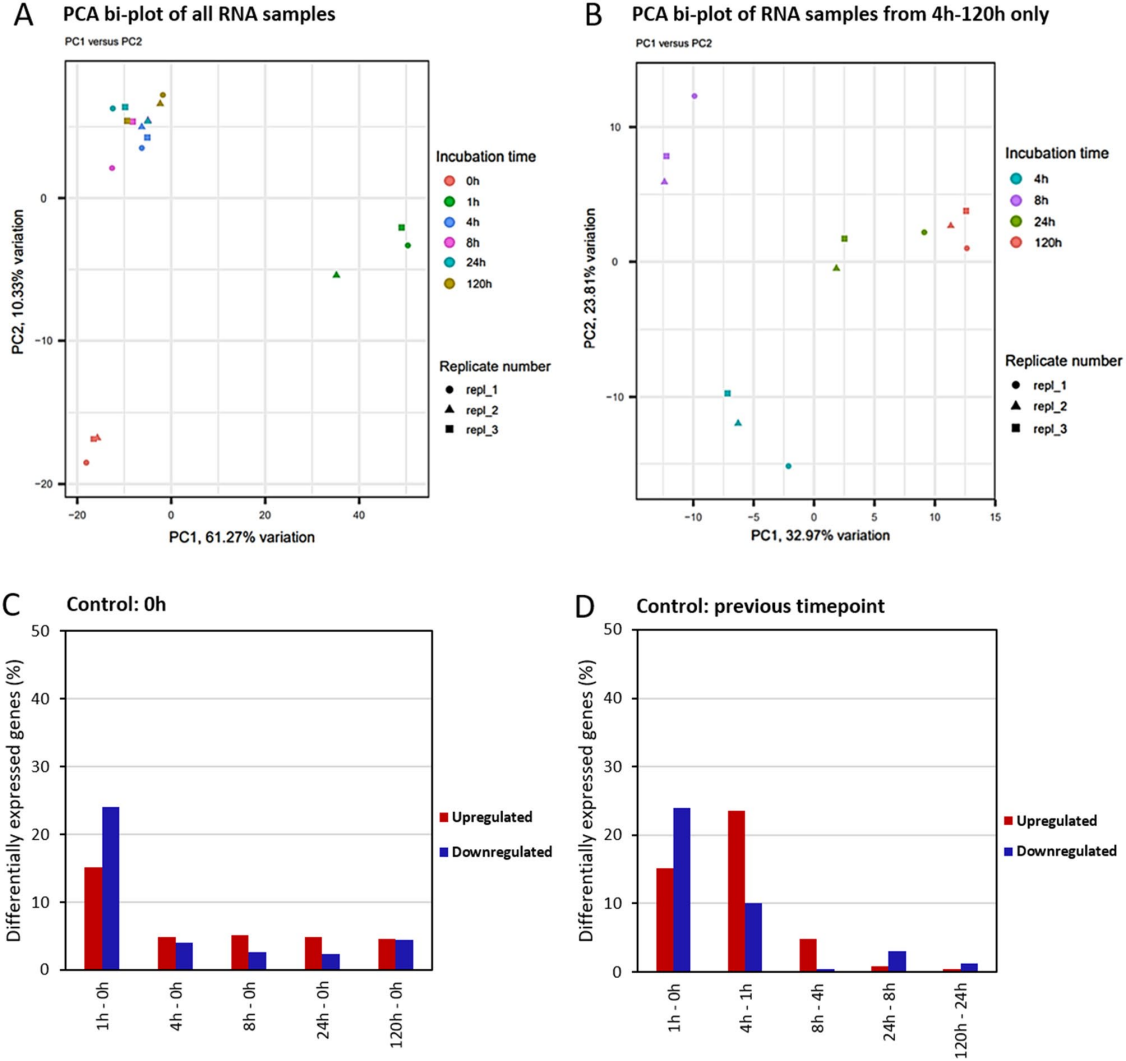
(**A**,**B**) PCA bi-plots of the RNA-Seq samples collected at 0 h, 1 h, 4 h, 8 h, 24 h and 120 h and for samples collected at 4 h, 8 h, 24 h and 120 h after the increase in temperature from 30 to 42 °C. The first two PCs are displayed. (**C**,**D**) Percentage of DEGs over time, observed in *Picochlorum* sp. (BPE23) after an increase in temperature from 30 to 42 °C. The total pool of genes was 6990. The time after temperature shift is displayed on the x-axis, as is the sampling time that was used as control. Up-regulated genes are indicated in green whereas down-regulated DEGs are indicated in red. DEGs were selected based on an FDR-adjusted *p* value ≤ 0.05 and an absolute log2 fold change value > 1.

**Figure 5:**
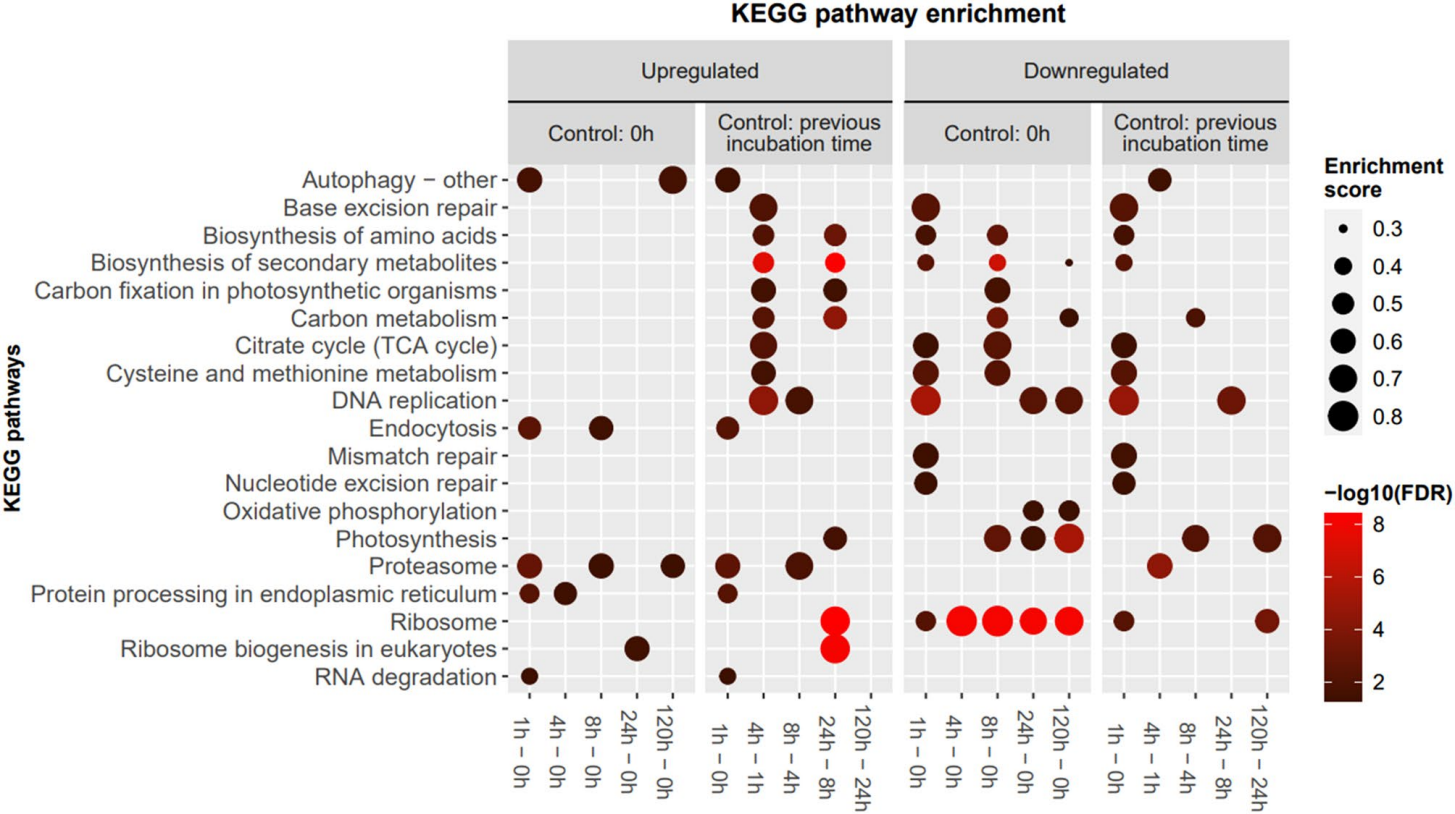
Pathway enrichment analysis of the mRNA expression response of *Picochlorum* sp. (BPE23) at each cultivation time after an increase in temperature from 30 to 42 °C. The dot size indicates the enrichment score of the KEGG pathway whereas the color brightness indicates significance (− log_10_(FDR)).

**Figure 6:**
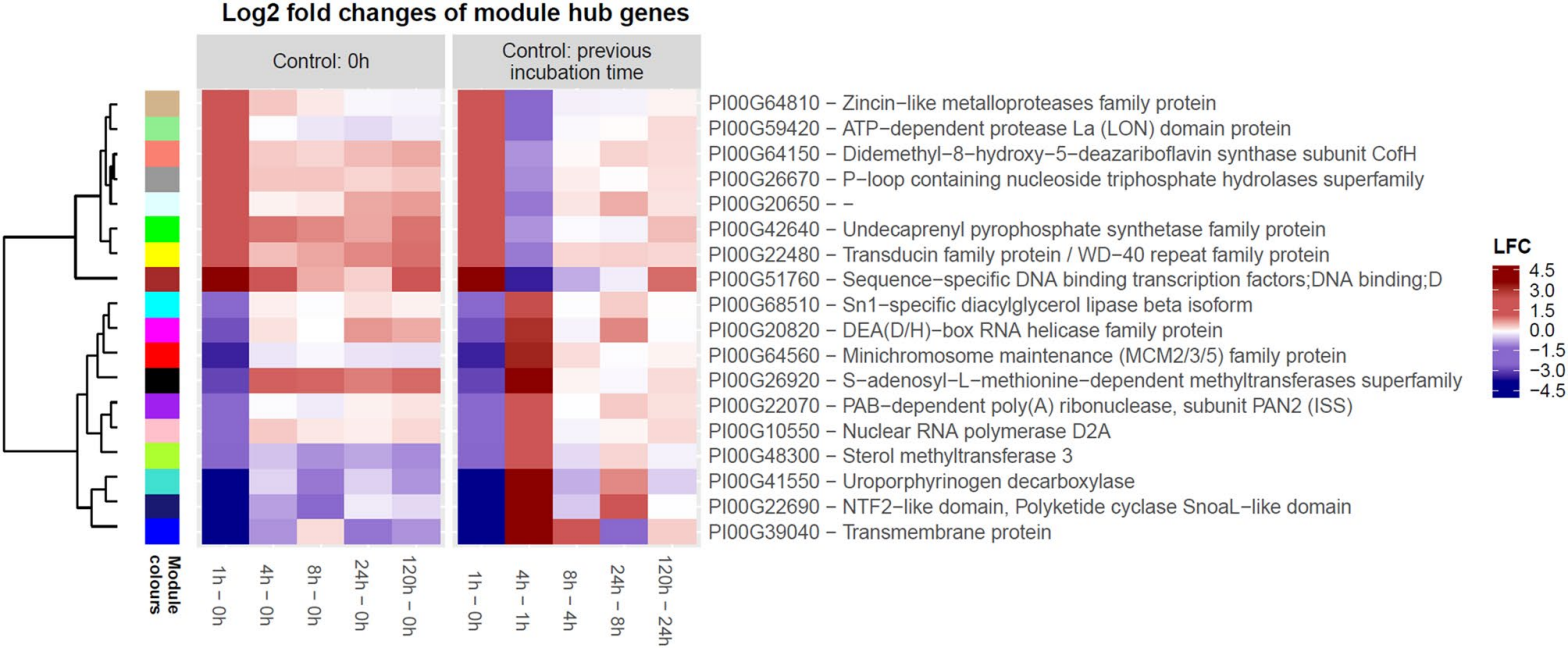
Module hub genes associated with processes affected by temperature stress in *Picochlorum* sp. (BPE23). Modules are defined from network inference by WGCNA of genes in the temperature-shock experiment. A heatmap represents differential expression levels of mRNA. Module color is displayed on the left. LFC represents the log2 fold change.

Differential gene expression levels and enrichment analyses are displayed in two ways in Figs. 4C,D, 5, and 6. The left-hand side of Fig. 4 shows the number of genes that are differentially expressed in the individual timepoints compared to the control condition at timepoint zero, which represents steady state growth at 30 °C. The right-hand side of the figure shows the number of genes that are differentially expressed between consecutive timepoints.

39% of all genes showed an log2 fold change value larger than 1 after 1 h, which was largely reversed between 1 and 4 h (Fig. 4). The number of differentially expressed genes (DEGs) decreased from 4 to 8 h, and subsequently to 24 h after the temperature increase. This indicates that the initial heat stress subsided and that *Picochlorum* sp. (BPE23) was reverting to a stable state once again. Between 24 and 120 h only 2% of genes were differentially expressed. The changes in log2 fold change after 8 h, 24 h and 120 h look very similar when compared to 0 h. However, when compared to the previous sampling time then differences in expression levels can be observed.

A PCA was conducted to identify similarities among biological replicates (Fig. 4). mRNA expression at 0 h and 1 h showed a significant distance from samples taken at 4 h, 8 h, 24 h, and 120 h. A second PCA was then done using only samples at 4 h, 8 h, 24 h, and 120 h. The samples taken at 4 h, 8 h, 24 h, and 120 h cluster together by biological triplicate and display distance to sample moments as expected. The clustering of biological replicates confirms that samples were processed and sequenced correctly.

### Temperature stress affects protein processing, HSP expression, and DNA replication

KEGG pathway enrichment was performed to identify which metabolic pathways were affected by the temperature increase (Fig. 5). The RNA degradation pathway was upregulated directly after temperature increase. Other upregulated pathways involved in the immediate response were the proteasome, autophagy, and endocytosis pathways. Downregulated pathways were DNA replication, ribosome, and base/nucleotide excision repair. In addition, pathways in the central metabolism such as biosynthesis of amino acids, biosynthesis of secondary metabolites, and citrate cycle were downregulated 1 h after the temperature increase. A reversal of this initial downregulation was observed after 4 h. Despite the observed upregulation between sampling moments, the processes were never enriched in the upregulated pathways when compared to growth at 0 h (30 °C). The ribosome pathway remained enriched in the downregulated pathways compared to timepoint zero throughout the experiment. Photosynthesis pathway was downregulated after 8 h. Despite enrichment in the upregulated pathways at 24–8 h, the photosynthesis was still downregulated compared to 0 h. From 24 to 120 h after the temperature shift, downregulation increased further.

GO-term enrichment analysis was conducted in addition to the KEGG enrichment to further identify enriched cellular processes (Supplementary material 2). Various GO-terms related to protein processing such as Protein folding, Proteolysis and Proteasome were upregulated throughout the experiment. These GO-terms showed enrichment in the upregulated GO-terms compared to the control at 0 h. In addition, cellular response to heat and heat shock protein binding show enrichment in the upregulated GO-terms. On the contrary, DNA replication, photosynthesis, peptide biosynthesis, translation, and three ribosome related GO-terms are enriched in the downregulated genes. The observations from the GO-term enrichment indicate similar changes in the mRNA expression as the observations from the KEGG pathway enrichment indicated.

### Network analysis verifies the results of the KEGG enrichment analysis

Weighted gene co-expression network analysis (WGCNA) was performed to describe correlation patterns among genes. Genes were clustered into 18 modules based on co-expression patterns (Fig. 6, Supplementary material 3). Each module was functionally annotated with GO-terms and KEGG pathways to assess differences between modules (Supplementary material 4). Each module displayed a unique functional annotation pattern which consisted of multiple GO-terms. The hubs were grouped roughly by similarities in functionality. The different module colours and their phylogenetic relationships are displayed in Fig. 6. The brown, cyan, green, grey and yellow modules were made up of genes associated with proteolysis, protein ubiquitination, autophagy, endocytosis and protein processing in ER. These processes are involved in the protein-related protection response to temperature stress^4^. Furthermore, the light-green, black, green–yellow and turquoise modules were associated with processes to energy fixation and metabolism, such as ATP binding, oxidative phosphorylation, photosynthesis, thylakoid membrane, carbon fixation, glycolysis and gluconeogenesis. Other interesting modules which were affected by temperature stress are the blue, light-cyan, magenta, pink and red module. These modules are associated with RNA-, and DNA-related processes such as replication, binding and repair. Lastly, all hubs except for grey and yellow were associated to the chloroplast envelope, chloroplast stroma, and plastid.

The average module expression profile was summarised through the module’s eigengene. The eigengene is a hypothetical gene that is highly correlated with expression profiles of genes in the module and can therefore function to represent the average expression of the module^20^. Genes with the highest eigengene-based connectivity were selected as hub genes, and presented together with their expression levels (Fig. 6). The largest changes in differential expression were observed during the first hours of the experiment. The transcriptional response in the first hour after the temperature increase displayed the largest log2 fold change value, a large reversal was observed for all modules after 4 h. Despite this reversal in differential expression, about half of the hub genes stay up or downregulated to a lower extent throughout the experiment. Hub genes are representative for their associated hub and therefore these hubs and their associated functions, as annotated through GO-term and KEGG pathway enrichment, hypothetically display a comparable expression pattern (Supplementary material 4). In general, the results from the WGCNA correspond to enrichment analysis results. Several interesting hub genes were identified which play a role in the response to an increase in temperature.

## Discussion

### Cell physiology changes severely after exposure to supra-optimal temperature

In an industrial photobioreactor, temperature can raise to levels that significantly affect the growth and productivity of microalgae. Microalgae are continuously exposed to changing environmental conditions throughout the day and between days. We aimed to characterize the effect of supra-optimal temperature on the physiology of the green microalga *Picochlorum* sp. (BPE23). We subjected *Picochlorum* sp. (BPE23) to a supra-optimal temperature of 42 °C for 120 h, whereas 38 °C is the optimal temperature for growth^14^. Cell growth, cell volume, pigment composition, fatty acid composition and mRNA expression were measured throughout the experiment. While such long exposure to high temperature does not commonly occur in nature or in photobioreactors, this study generated knowledge on how microalgae immediately cope and eventually acclimate to supra-optimal temperatures.

Two different sequential response phases were observed after the temperature shift from 30 to 42 °C. In the first phase (0–8 h), the cell cycle was arrested and an increase in cell volume was observed. The cell cycle resumed after 8 h as cells started to divide and returned to their original cell size. During the second phase (8–120 h), *Picochlorum* sp. (BPE23) acclimated towards a new homeostatic phase. The average cell volume gradually increased between 24 and 120 h. This increase in cell size was unexpected as increased temperature commonly causes a decreased cell volume in other microalgal species to counteract the imbalance between catabolic and anabolic processes^1^. The growth rate and cell composition stabilized after 24–72 h. The changes in mRNA transcription were very severe between 0 and 4 h, while between 4 and 120 h differential expression levels rapidly became smaller.

Interestingly, the hub gene in the red module, minichromosome maintenance (MCM2/3/5) family protein, was downregulated 5 times log2 fold during the first hours after temperature increase. The MCM complex is critical for cell division and essential for DNA replication as it is a target of various checkpoint pathways required for S-phase entry^21^. This confirms the hypothesis that the cell cycle was arrested to protect the DNA from temperature-induced damage during the replication cycle. In literature, the cell cycle of *Chlamydomonas reinhardtii* was found to be inhibited after a heat shock treatment^4,12^. Full recovery of the cell cycle and recovery of the cell physiology in *C. reinhardtii* was only observed after the temperature was decreased.

### Heat shock proteins were upregulated directly after the temperature increase

A heat shock response was observed in the first hours, and to a lesser extent throughout the experiment. HSP20 and HSP70 were found among the strongest upregulated genes in the dataset. HSP70 was the main cause for enrichment in the endocytosis, protein processing and the endoplasmic reticulum pathways. Moreover, genes encoding for HSP40, HSP60, and HSP90, and HSFs showed upregulated gene expression. HSFs are transcriptional activators that regulate expression of heat shock proteins. The heat shock proteins protect cells from adverse effects of thermal stress through chaperoning and refolding of polypeptides and by stabilizing protein complexes, cellular membranes and key cellular processes^5,6,22^. Their regulatory role provides thermotolerance and the ability to gradually acclimate to a new environmental temperature by mediating the part of the cellular stress response that deals with protein homeostasis^23^. Next to the high upregulation of heat shock proteins, most of the other genes in the endocytosis pathway were also upregulated (~ 0.5–1 times log2 fold). In addition to regulation by heat shock proteins, the 5.3 times log2 fold upregulation of the ubiquitin-like superfamily protein-encoding genes is the major cause for the enrichment of the autophagy pathway and is involved in numerous stress-related functions such as the cell cycle, DNA repair, transcription, autophagy, and post-translational protein modification^24^. Proteins can still become damaged despite chaperoning and stabilization. Degradation of damaged proteins and proteins that are no longer required for cell functioning is essential to maintain cellular homeostasis^2^. The 20S proteasome alpha subunit F2 (PAF2) encoding gene was upregulated 7.5 times log2 fold and is involved in the degradation of proteins with partially unfolded regions^25^.

DNA repair in microalgae is still poorly understood^2,6^. However, heat shock proteins are known to initiate DNA repair after stressful events. HSP70 and HSP27 in particular are associated to this activity^26^. DNA replication, base excision repair, Nucleotide excision repair, and mismatch repair were enriched in the downregulated genes at 1 h, whereas DNA replication was enriched in the downregulated genes at 24 h and 120 h. The DNA associated network interference module (red) shows a similar pattern of downregulation, followed by almost complete reversal towards a log2 fold change value of ~ 0. Hypothesizing from the mRNA expression data, DNA was not damaged after exposure to supra-optimal temperatures, nor by formation of ROS.

### Supra-optimal temperature downregulates photosynthesis but increases pigment concentrations.

The quantum yield (F_v_/F_m_) increased during the first hours after the temperature increase, indicating an increased efficiency of photosystem II at 42 °C. After 8 h, the quantum yield decreased until the end of the experiment. Moreover, between 8 and 120 h, gene expression of nuclear genes encoding subunits of both photosystem I and photosystem II was enriched in the downregulated genes. mRNA expression for genes associated to both photosystems was downregulated despite the fact that photosystems are not considered a bottleneck at survivable but supra-optimal culture temperatures. In addition, several genes in the *Porphyrin and chlorophyll metabolism* show up to fourfold downregulation, of which the most severely downregulates genes are: Coproporphyrinogen III, Uroporphyrinogen decarboxylase, and NAD(P)-binding Rossman-fold superfamily protein. Genes enriched in the turquoise hub, associated with chloroplasts, plastids, and the thylakoid membrane, showed downregulation throughout the experiment. Research on *Dunaliella bardawil* reported that photosynthesis was severely downregulated and that chlorophyll content decreased dramatically after exposure to a heat treatment of 42 °C for 2 h, after which temperature was returned to the optimal temperature^13^. In *Picochlorum sp. (BPE23)*, the mRNA transcript levels indicate overall decreased photosynthetic activity. However, an unexpected increase in chlorophyll-a and chlorophyll-b was observed nonetheless.

Downregulation of photosynthesis is done to reduce the formation of ROS^13^. When metabolism becomes unbalanced due to temperature stress the excessive energy flux from photosynthesis results in the formation of ROS. Unfortunately, too few genes in the carotenoid biosynthesis KEGG pathway gained a functional annotation. Therefore, little insight on the transcriptional response of the carotenoid biosynthesis pathway was gained. However, data on pigment concentration in the biomass show that the concentration of the carotenoids: zeaxanthin, violaxanthin, lutein, β-carotene and canthaxanthin increased as a result of exposure to supra-optimal temperature. An increase in carotenoid content in response to temperature increase was also found in the literature for *D. bardawil* and *Haematococcus pluvialis*^13,27^. Increased levels of carotenoids are often induced when microalgae are exposed to abiotic stress conditions such as salinity, light, and temperature for quenching of ROS^10,28^.

### The metabolism and protein synthesis were downregulated to conserve energy

As expected, connected to downregulation of genes associated to photosynthesis, the carbon fixation pathway and central energy metabolism were downregulated as determined through KEGG pathway enrichment. All analyses point towards downregulation of the metabolism of *Picochlorum* sp. in response to temperature stress. Lower photosynthetic activity ultimately results in depletion of the cell’s energy reserves. Suppression of ribosome biogenesis reduces the energy demand of the cell through reduction of protein synthesis to conserve energy^25^. Ribosomal activity was significantly downregulated throughout the experiment. In a study with a thermosensitive *Arabidopsis Thaliana* strain it was hypothesized that the ribosomal activity was downregulated in response to stressful temperature to prevent errors in translation and protein folding^29^. Production of misfolded proteins would not only lead to wasted energy, but could also lead to formation of toxic compounds in the cell that cause further damage^8^. Therefore, reduced ribosomal activity indirectly relieves the workload of chaperones. Other protein processing related processes were affected throughout the experiment. The rate of protein synthesis was adapted to the reduced growth rate that was caused by the increased temperature. At the same time, the conserved energy was available for damage repair caused by oxidative stress and acclimation to the new temperature^6^. Downregulation of protein production and photosynthesis after temperature stress was also observed in *Picochlorum costavermella*^29^. We observed that downregulation of these vital cellular processes led to a severe decrease in growth rate.

### *Picochlorum* sp. (BPE23) accumulated fatty acids under heat stress

The concentration of neutral fatty acids is initially low (3.0 mg g^−1^) as cells are grown in nitrogen replete growth medium^30^. However, 24 h after temperature increase, a fatty acid concentration of 11.5 mg g^−1^ was measured. This significant 383% increase shows the funnelling of excess energy into de novo synthesis of lipid bodies as energy storage. In a study on *Chlamydomonas reinhardtii* and *Chlorella vulgaris* a screening was done for neutral lipid accumulation at elevated temperature, after which about 25% of the mutant strains showed similar elevated lipid accumulation^31^. Apparently, induction of neutral lipids through increased temperature is strain specific. Next to neutral lipids, also the polar membrane fatty acid concentration increased, from 79.0 to 88.5 mg g^−1^. Especially C16:0 increased significantly. A study on *Chlamydomonas reinhardtii* showed that translocation of unsaturated fatty acids from cell membranes to neutral lipids happened after a temperature increase, to maintain optimal membrane fluidity^4^. Especially plastid membranes are heat sensitive, and quick modification of these membranes is necessary to keep photosynthesis running^32^. Almost all hubs found through the WGCNA analysis show gene ontology annotation to the plastid membranes, indicating that these membranes are also severely affected in *Picochlorum* sp. (BPE23).

Genes in the fatty acid biosynthesis pathway exhibited downregulation as an overall trend from 0 to 1 h, although the pathway itself was not significantly enriched due to the presence of few severely upregulated genes, up to 2.6 times log2 fold (Supplementary material 6). Pathview pathway analysis indicated increased mRNA expression of genes encoding for Malonyl-CoA synthase, which catalyses the reaction from malonate into malonyl-CoA. In addition expression of mRNA of acyl carrier protein and long chain acyl-CoA synthase were upregulated. However, drawing accurate conclusions is challenging as the fatty acid biosynthesis pathway is only partly annotated in the used reference genome of *Picochlorum* sp.* SENEW3* (assembly ASM87641v1)^33^. Another possible reason for the fatty acid accumulation could be high stability of enzymes involved in fatty acid biosynthesis which resulted in the continuation of fatty acid biosynthesis, whereas other metabolic activity and cell growth decreased. Although the total increase in fatty acid content was just 18 mg g^−1^ at 24 h and 24 mg g^−1^ at 72 h, this increase corresponds to a percentual gain in fatty acid content of 22% and 38%, respectively. The ability to induce an increased fatty acid concentration by increasing temperature for only 24 h opens up the possibility to boost the lipid content of microalgal biomass before harvest.

## Conclusions

In this study, the mechanisms underlying temperature stress and acclimation were determined through assessment of cell physiology and transcriptome analysis. A cell cycle arrest and a transcriptomic heat shock response were observed directly after an increase in temperature to 42 °C, 4° above the optimal growth temperature. During the first 8 h, various regulatory and chaperoning mechanisms were differentially expressed to protect the cells from heat-induced cell damage. Between 8 and 120 h after temperature increase, an acclimation process towards a new homeostatic phase started. Photosynthesis, carbon fixation, ribosomal activity, and central metabolism were downregulated, hypothetically to reduce oxidative cell damage. At the same time, pigment concentration increased significantly and the concentration of fatty acids increased by 22% over between 0 and 24 h. The combination of these effects led to a decrease in growth rate throughout the experiment. This study contributes knowledge on the effect of supra-optimal temperature on the physiology of the industrially relevant green microalgae *Picochlorum* sp. (BPE23).

## Materials and methods:

### Cell cultivation

#### Growth media and inoculum preparation

*Picochlorum* sp. (BPE23), isolated from a saltwater body of Bonaire was pre-cultivated in shake flasks in an orbital shaker incubator (Multitron, Infors HT) with a 12/12 h day/night cycle and a light intensity of 100 μmolph m^−2^ s^−1^^14^. The temperature was 30 °C during night and 40 °C during day. Furthermore, the relative humidity of the air in the incubator was set to 60% and enriched with 2% CO_2_. Cells were cultured in artificial seawater enriched with nutrients and trace elements. Elements were provided at the following concentrations (in g L^−1^): NaCl, 24.5; MgCl_2_.6H_2_O, 9.80; Na_2_SO_4_, 3.20; NaNO_3_ 2.12; K_2_SO_4_, 0.85; CaCL_2_·2H_2_O, 0.80; KH_2_PO_4_, 0.23; Na_2_EDTA·2H_2_O, 0.105; Na_2_EDTA, 0.06; FeSO_4_·7H_2_O, 0.0396; MnCl_2_·2H_2_O, 1.71 × 10^−3^; ZnSO_4_·7H_2_O, 6.60 × 10^−4^; Na_2_Mo_4_·2H_2_O, 2.42 × 10^−4^; Co(NO_3_)_2_·6H_2_O, 7.00 × 10^−5^; NiSO_4_·6H_2_O, 2.63 × 10^−5^; CuSO_4_·5H_2_O, 2.40 × 10^−5^; K_2_CrO_4_, 1.94 × 10^−5^; Na_3_VO_4_, 1.84 × 10^−5^; H_2_SeO_3_, 1.29 × 10^−5^. HEPES (4.77 g L^−1^) was added for shake flask cultures as a pH buffer. The medium pH was adjusted to 7.4 after and filter sterilized before use. During photobioreactor cultivation, Antifoam B (J.T.Baker, Avantor, USA) was added at a concentration of 0.5 mL L^−1^ out of a 1% w/w% stock. In addition, 0.168 g L^−1^ Sodium bicarbonate (NaHCO_3_) was added at the time of inoculation to provide sufficient CO_2_ at the start of the cultivation. The photobioreactor was inoculated at a starting OD density of 0.2.

#### Photobioreactor operation

Microalgae were cultivated in heat-sterilized flat panel photobioreactors (Labfors 5 Lux, Infors HT, Switzerland) with a working volume of 1.8 L, an optical depth of 20.7 mm and a surface area for irradiation of 0.08 m^2^. Continuous irradiation (24/24 h) was applied from one side by 260 warm white LED lamps at 813 μmol_ph_ m^−2^ s^−1^ (PAR). To remove variation in gene expression due to the circadian cycle we grew the microalgae under continuous irradiation while maintaining all other growth conditions stable at the same time. The biomass density was maintained at approximately 2.3 g L^−1^ by continuous light controlled dilution of the cell culture (turbidostat mode). The turbidostat control was set to maintain an outgoing light level of 10 μmol_ph_ m^−2^ s^−1^ (PAR). The dilution rate of the photobioreactor was logged continuously by weighing the ingoing medium vessel. Compressed air was supplied at a rate of 980 mL min^−1^. CO_2_ was provided on-demand by pH-controlled addition. The pH level in the photobioreactor was set at 7. The photobioreactor temperature was set at 30 °C from the start of cell cultivation. When steady state was reached, the temperature was increased to 42 °C in one step, which took approximately 15 min. Samples were taken daily at 0 h, 1 h, 4 h, 8 h, 24 h after the temperature increase, and once a day for every day onwards to monitor changes in cell physiology.

### Biomass analysis

#### Dry weight

Biomass concentration (g L^−1^) was measured in duplicate by dry weight determination. Empty Whatman glass microfiber filters (θ 55 mm, pore size 0.7 μm) were dried overnight at 95 °C and placed in a desiccator for 2 h. Filters were then weighed and placed in the mild vacuum filtration setup. Cell culture containing 1–10 mg of microalgae biomass was diluted in 25 mL 0.5 M ammonium formate and filtered. The filter was washed twice with 25 mL 0.5 M ammonium formate to remove residual salts. The wet filter was dried overnight at 95 °C, placed in a desiccator for 2 h, and weighed. Biomass concentration was calculated from the difference in filter weight before and after filtration and drying.

#### Cell volume and number

Cell size and cell number were measured in duplicate with the Multisizer III (Beckman Coulter Inc., USA, 50 μm aperture). Samples were diluted in two steps before analysis, initially by dilution of 5× in fresh medium, followed by dilution of 100× in Coulter Isoton II. Cell volume was then derived from the cell size by assuming that cells were shaped spherical.

#### Quantum yield

The cell culture’s quantum yield (F_v_/F_m_), representing the maximum photosynthetic capacity of photosystem II was determined. Cells were measured after dark adaption at room temperature for 15 min (AquaPen-C 100, PSI; excitation light 455 nm (blue), saturating light pulse: 3000 µmol m^−2^ s^−1^).

#### Biomass harvest and lyophilizing

Biomass samples for compositional analysis were taken at the same moment as when offline measurements were performed. Microalgae cells were pelleted by centrifugation at 4000*g* for 5 min and washed with 0.5 M ammonium formate. The centrifugation/washing cycle was repeated twice more after which the cell pellet was frozen at − 20 °C. Samples were then lyophilised for 24 h and stored at − 20 °C until further processing.

#### Pigment analysis

Pigment content was determined through extraction and HPLC analysis ^34^. 10 mg of lyophilized biomass was disrupted by bead beating (Precellys 24, Bertin Technologies, France) at 5000 rpm for three cycles of 90 s with 60-s breaks on ice between each cycle. The extraction was done through five washing steps with methanol containing 0.1% butylhydroxytoluene. Separation, identification and quantification of pigments were performed using a Shimadzu (U)HPLC system (Nexera X2, Shimadzu, Japan), equipped with a pump, degasser, oven (25 °C), autosampler, and photodiode array (PDA) detector. Separation of pigments was achieved using a YMC Carotenoid C30 column (250 × 4.6 mm 5 μm ID) coupled to a YMC C30 guard column (20 × 4 mm, 5 μm ID)(YMC, Japan) at 25 °C with a flow rate of 1 mL min^−1^. A sample injection volume of 20µL was used. The mobile phases consisted of Methanol (A), water/methanol (20/80 (v/v%)) containing 0.2% ammonium acetate (B), tert-methyl butyl ether (C) (all solvents were purchased at Sigma Aldrich). The elution protocol started with 0–12 min isocratic A:95% B:5% C:0%, with at 12 min a step to A:80%, B:5%, C:15%, followed by a linear gradient 12–30 min to A:30%, B:5%, C:65%, finally followed by a conditioning phase 30–40 min at the initial concentration. Analytical HPLC standards for chlorophyll a, chlorophyll b, β-carotene, canthaxanthin, violaxanthin, antheraxanthin, zeaxanthin, and lutein had a purity of > 99% (Carotenature, Switzerland).

#### Fatty acid analysis

Fatty acids within the triacylglycerol (TAG) and polar lipids (PL) fraction were quantified through GC-FID analysis according to^35^. 10 mg of lyophilised biomass was disrupted by bead beating. The fatty acids were extracted from the disrupted biomass in a mixture of chloroform/methanol (1:1.25, v:v) containing Glyceryl tripentadecanoate (C15:0 TAG) (T4257, Sigma-Aldrich) and 1,2-didecanoyl-sn-glycero-3-phospho-(1′-rac-glycerol) (sodium salt) C10:0 PG (840434, Avanti Polar Lipids Inc) as internal standards for the TAG and the PL fraction, respectively. Separation of TAG and PL was done by use of Sep-Pak Vac silica cartridge (6 cc, 1000 mg; Waters). TAG was eluted from the column with a solution of hexane:diethylether (7:1, v:v) and the PL were eluted with a solution of methanol:acetone:hexane (2:2:1, v:v:v). The extracts were methylated for 3 h at 70 °C in methanol containing 5% H_2_SO_4_.

### Transcriptome analysis

#### mRNA extraction and sequencing

mRNA-Seq analysis was done for samples taken at 0 h, 1 h, 4 h, 8 h, 24 h and 120 h after the temperature increase from 30 to 42 °C. Biomass was directly put on ice and centrifuged for 5 min at 4255 g at 2 °C. The cell pellet was then immediately frozen in liquid nitrogen and stored at − 80 °C until further processing. mRNA was extracted from ~ 200 ul frozen cell pellet by automated mRNA extraction (Maxwell® 16 LEV simplyRNA, Promega, USA). Extracted mRNA was tested for integrity (Qsep100, GCbiotech, Netherlands) and quantity (Qubit fluorometer, ThermoFisher, USA). Sequencing libraries were prepared using the NEB Next® Ultra™ mRNA Library Prep Kit. Fragments of the mRNA library in the size range of 250–300 bp were sequenced using the Illumina Novoseq PE150 platform, yielding paired-end reads of 150 nt (Novogene, China). The quality of mRNA-Seq reads was assessed using FastQC v0.11.5 ^36^.

#### Transcript assembly and annotation

Paired-end reads were mapped to the genome of *Picochlorum* sp.* SENEW3* (assembly ASM87641v1) using HISAT2 v2.2.1 with the -very-sensitive pre-set ^33,37^. Transcript were assembled and predicted using StringTie v2.1.4, which was guided by the structural annotation of *Picochlorum* sp. *SENEW3*. StringTie’s prepDE3 python script was used to extract gene counts and predict genes de novo. Functional annotation was initiated by a BLASTP search of the translated *Picochlorum* sp. (BPE23) coding sequences against the protein sequences of *Arabidopsis thaliana* with an E-value threshold of 1E^−10^. Unannotated genes were filtered and orthology inference was conducted using OrthoFinder v2.5.2 against the protein sequences of the microalgae *Auxenochlorella protothecoides*, *Chlorella variabilis*, *Chlamydomonas reinhardtii*, and *Helicosporidium* sp. Ortholog gene identifiers were then matched to their gene description. Functional annotation was concluded by matching unannotated genes to their inferred domains, as derived from Pico-PLAZA.

#### Differential expression analysis and GO, and KEGG enrichment analysis

Pairwise differential expression (DE) analysis was performed using the DESeq2 v1.30.0. R package. Sample-level quality control consisted of pairwise correlation clustering, hierarchical clustering, and Principal Component Analysis (PCA). Fold change values were generated on a log2 scale (LFC). Two designs for data display were implemented; first, where a control condition after 0 h was used for each sample to compare DE between stressed and non-stressed growth. Second, where the previous sampling moment was used as a control condition to compare DE over time. Genes with a false discovery rate (FDR) adjusted *p* value ≤ 0.05 and an LFC > 1 were considered as significantly differentially expressed.

*Arabidopsis thaliana* gene identifiers that matched to *Picochlorum* sp. (BPE23) genes were linked to their corresponding DE-analysis results and used for GO and KEGG pathway enrichment analysis^38^. Enrichment analyses were conducted by use of clusterProfiler v3.18.1. and org.At.tain.db v3.12.0. packages. GO-terms and KEGG pathways with an FDR-adjusted *p* value ≤ 0.05 and a positive or negative enrichment score were considered as significantly enriched and visualized with the ggplots2 package.

#### Network interference analysis

Weighted gene co-expression network analysis (WGCNA) was conducted by applying the WGCNA R package v1.69^20^. The correlation network was inferred from a correlation matrix of normalized counts. The optimal value of power was determined through scale-free topology analysis. The network was restricted to genes with informative connectivity, referring to a connectivity higher than the median connectivity of the entire network. Modules were then constructed with average linkage hierarchical clustering using distances in the topological overlap construction. Networks were constructed in a hybrid adaptive tree with a deepSplit of 1, a power of 12, a minimum cluster size of 30, a cut height of 0.8, and no PAM-like stage filtering. Subsequently, the gene with the highest eigengene-based connectivity was identified for each module and considered the module’s hub gene. Furthermore, modules were annotated with GO-terms and KEGG pathways to infer functional properties.

## Supplementary Information

Supplementary Information 1.

Supplementary Information 2.
