# Supplementary material for: Short-term physiologic response of the green microalga Picochlorum sp. (BPE23) to supra-optimal temperature
Source: Sci Rep. 2022 Feb 28;12:3290. doi: 10.1038/s41598-022-06954-6 (PMC8885816; doi:10.1038/s41598-022-06954-6)
Supplement: Supplementary file 2 — Supplementary Information 2. [file 41598_2022_6954_MOESM2_ESM.docx]

# Supplementary information files

#### Supplementary material 1

See attached excel file with the transcriptomic expression dataset named as: Supplementary material 2.xlsx

#### Supplementary material 2

**Supplementary figure 1**: GO enrichment analysis of the response of Picochlorum sp. (BPE23) after an increase in temperature from 30°C to 42°C at different moments during the experiment. The time after the temperature shift is displayed on the x-axis, the control condition is displayed above the x-axis. GO-terms are depicted in the ontologies Biological Process (BP), Cellular Component (CC) and Molecular Function (MF). The dot size indicates the enrichment score of the GO-term whereas the color brightness indicates significance (-log10(FDR)).


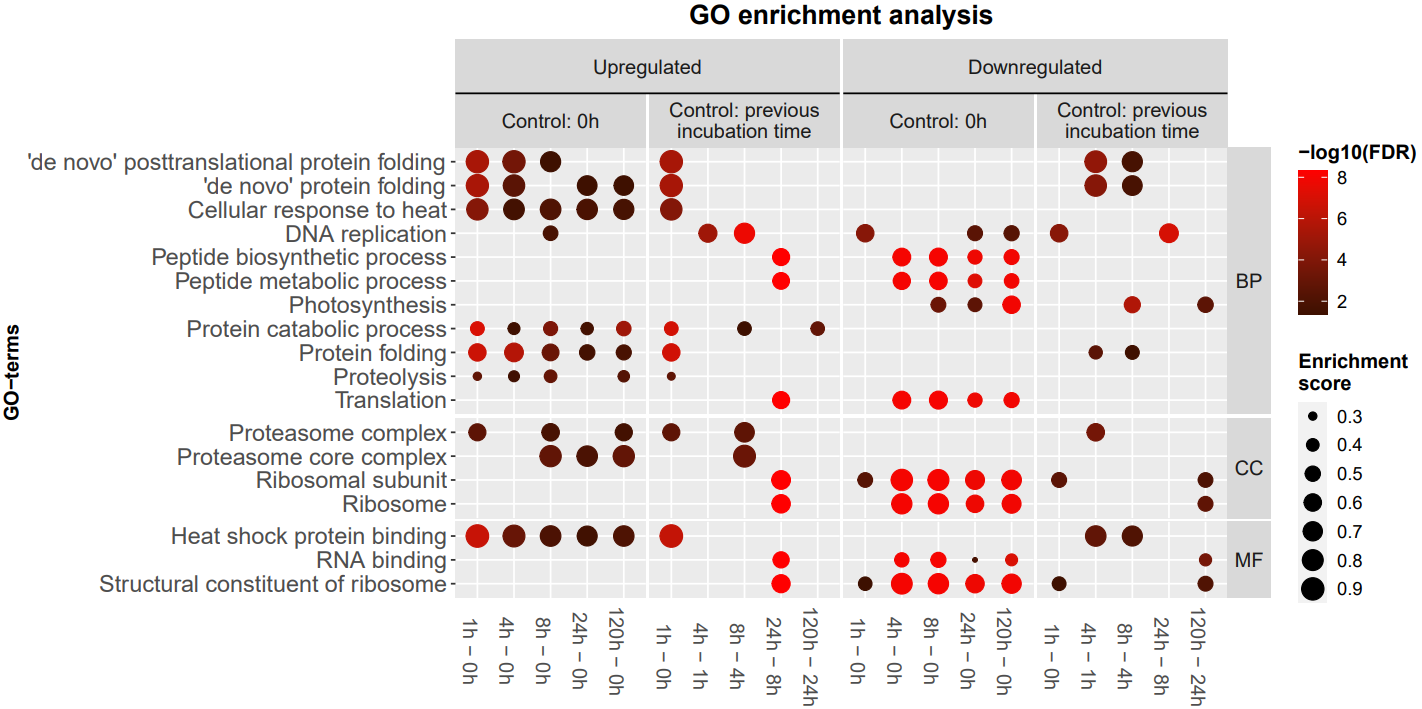


#### Supplementary material 3

Genes in the temperature-shock experiment were divided into eighteen modules based on co-expression patterns (Supplementary figure 3).

***Supplementary figure 2****: hierarchical dendrogram and module colors of the network tree as defined by WGCNA of genes in the temperature-shock experiment. The hybrid network tree was constructed by the Dynamic Tree Cut package with a minimum module size of 30, a cut height of 0.8 and a deep split (sensitivity) of 1 out of 3.*


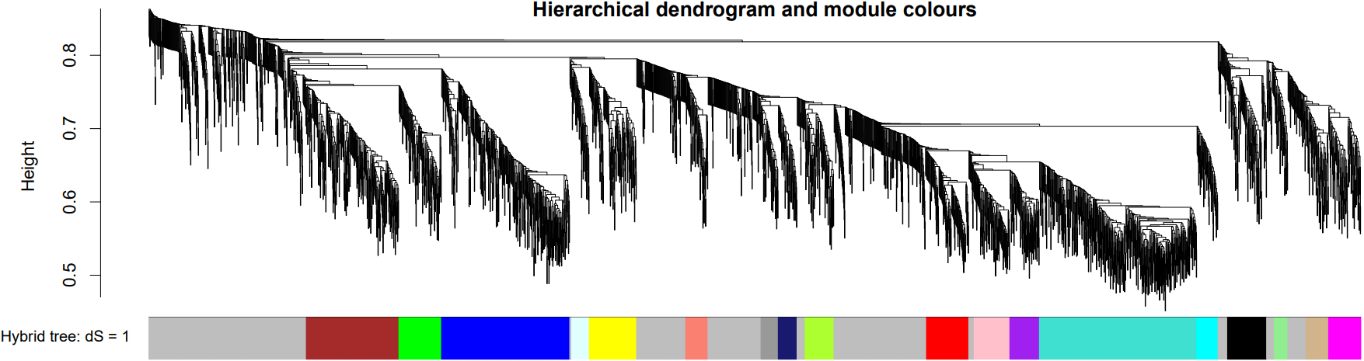


Genes with the highest kME were selected and considered hub genes. The kME and functional annotations of module hub genes are displayed in Supplementary table 1.

**Supplementary table 1:** The module, module size, eigengene-based connectivity (kME), gene ID, gene description, type of results used to infer the description and KEGG pathway annotations of hub genes. Hub genes are defined from network inference by WGCNA of genes in the temperature-shock experiment. Unknown information is indicated with a dash.

| Module | Module size | kME | Gene ID | Gene description | Result type | KEGG pathway annotation |
| --- | --- | --- | --- | --- | --- | --- |
| Black | 102 | 0.994 | PI00G26920 | S-adenosyl-L-methionine-dependent methyltransferases superfamily protein | BLASTP result | - |
| Blue | 334 | 0.996 | PI00G39040 | Transmembrane protein | BLASTP result | - |
| Brown | 241 | 0.993 | PI00G51760 | Sequence-specific DNA binding, TF, DNA binding | BLASTP result | - |
| Cyan | 55 | 0.990 | PI00G68510 | Sn1-specific diacylglycerol lipase beta isoform | Orthofinder result | - |
| Green | 111 | 0.990 | PI00G42640 | Undecaprenyl pyrophosphate synthetase family protein | BLASTP result | Terpenoid backbone biosynthesis |
| Green-yellow | 75 | 0.988 | PI00G48300 | Sterol methyltransferase 3 | BLASTP result | Steroid biosynthesis |
| Grey60 | 45 | 0.992 | PI00G26670 | P-loop containing nucleoside triphosphate hydrolases superfamily protein | BLASTP result | - |
| Magenta | 84 | 0.990 | PI00G33630 | DEA(D/H)-box RNA helicase family protein | BLASTP result | Spliceosome |
| Pink | 93 | 0.995 | PI00G10550 | Nuclear RNA polymerase D2A | BLASTP result | - |
| Red | 110 | 0.995 | PI00G46300 | Minichromosome maintenance (MCM2/3/5) family protein | BLASTP result | DNA replication |
| Turquoise | 410 | 0.997 | PI00G41550 | Uroporphyrinogen decarboxylase | BLASTP result | Porphyrin and chlorophyll metabolism |
| Yellow | 123 | 0.986 | PI00G42420 | Transducin family protein / WD-40 repeat family protein | BLASTP result | - |

#### Supplementary material 4

WGCNA was conducted for describing correlation patterns among genes in the temperature-shock experiment. The network modules were functionally annotated with GO-terms (Supplementary figure 4), and KEGG pathways (Supplementary figure 5).


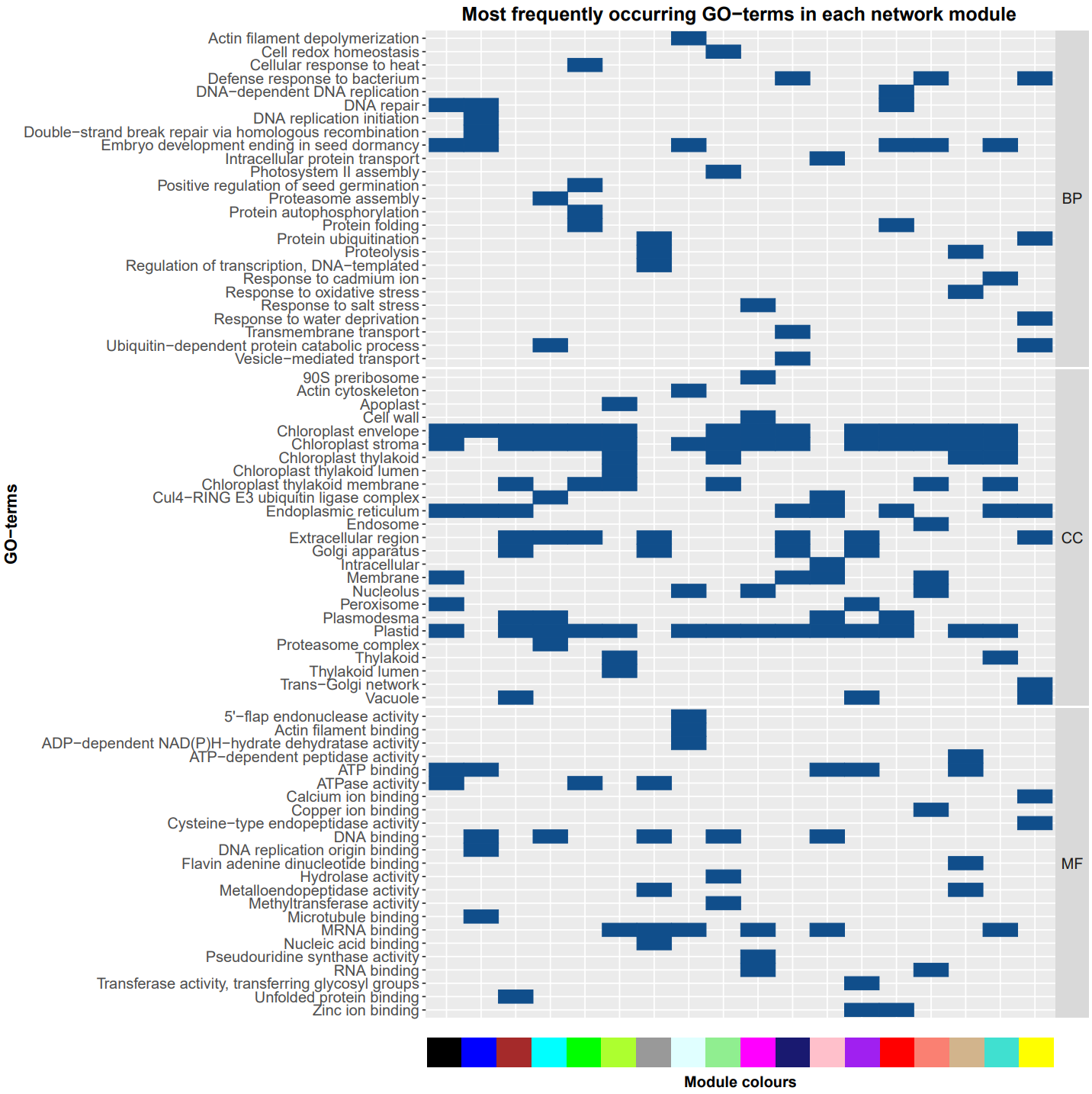


**Supplementary figure 3:** The ten most frequently occurring GO-terms in the ontologies Biological Process (BP), Cellular Component (CC) and Molecular Function (MF) in each network module, as defined by WGCNA analysis.


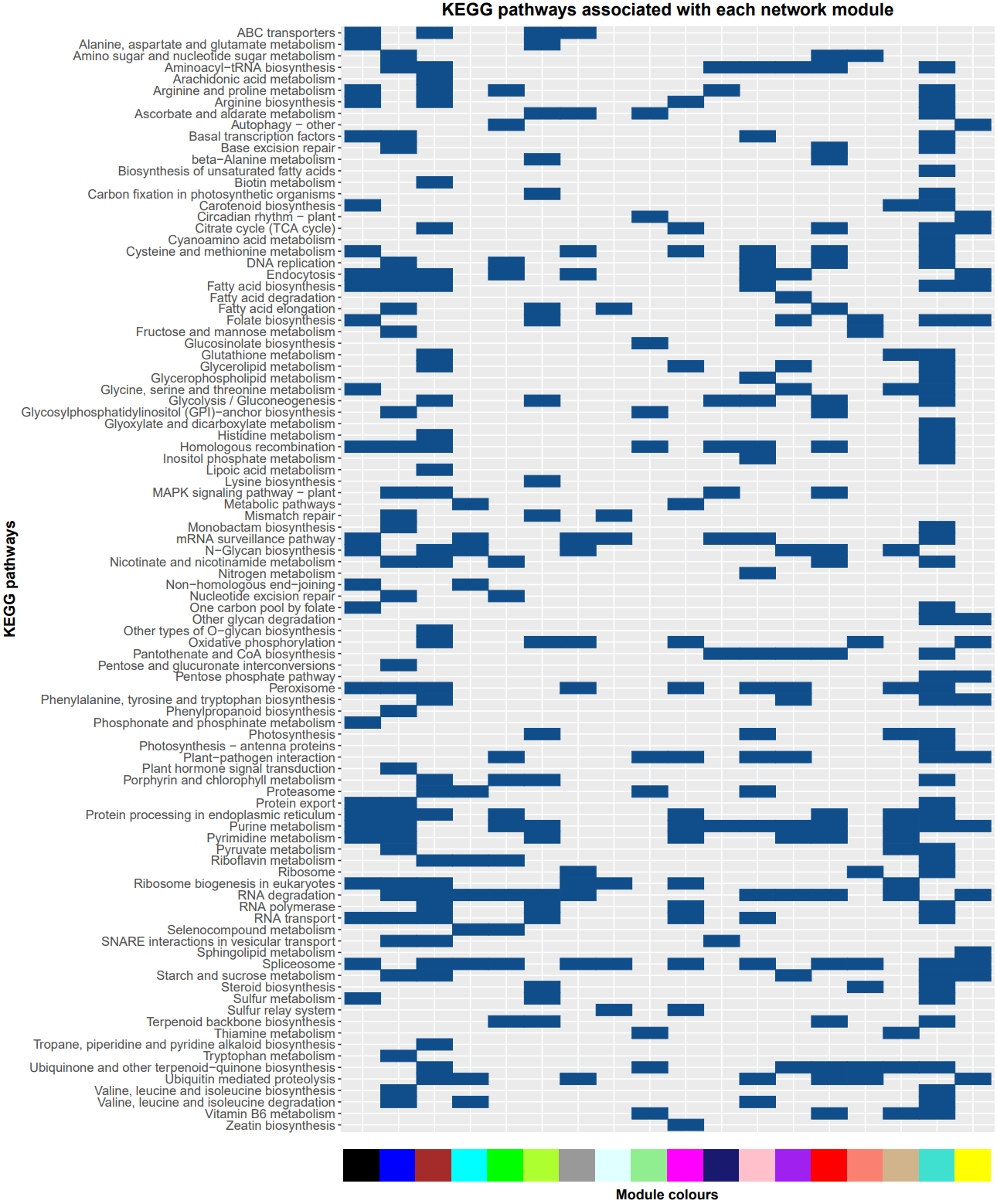


**Supplementary figure 4:** KEGG pathway annotations of each network module, as defined by WGCNA analysis

#### Supplementary material 5

The relative absorption of carotenoids (480 nm) versus biomass (750 nm) and the relative absorption of chlorophylls (680 nm) versus biomass (750 nm) were calculated and displayed in Supplementary figure 6. A quick decrease was observed during the first hours after the temperature increase after which a increasing trend was observed until 72 hours after the temperature step..

Optical density measured at 480, 680, and 750 nm can be used to qualitatively determine carotenoid, chlorophyll, and biomass content, respectively. The normalized 480/750 nm and 680/750 nm ratios display increased pigment content over time. This qualitative analysis agrees with the results of the quantitative pigment analysis, displayed in Fig 2.


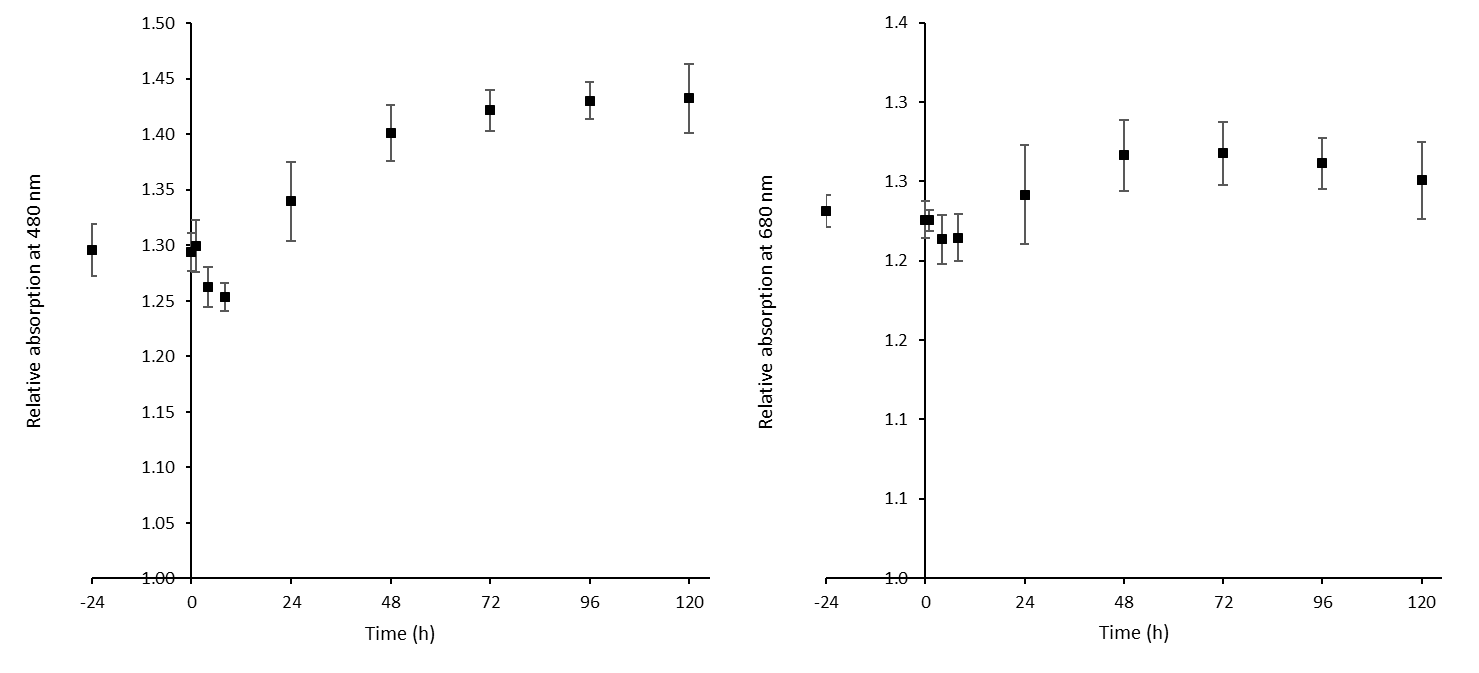


**Supplementary figure 5**: Relative absorption values of carotenoids and chlorophylls in biomass over time.

#### Supplementary material 6

Several genes from the Fatty acid biosynthesis pathway showed significant differential expression. Three of the most downregulated genes are coding for Thioesterase superfamily protein, NAD(P)-binding Rossman-fold superfamily protein, and 3-ketoacyl carrier protein synthase I and III, with a log2 fold change (LFC) of -3.1, -3.0, and -2.3 fold, respectively. Opposite, the genes encoding for Polyketide synthase enoyl reductase, acyl-activating enzyme 15, and plant stearoyl-acyl-carrier-protein desaturate were upregulated 2.6, 1.6, and 1.7 fold, respectively.

**Supplementary figure 6**: Pathview analysis for the Fatty acid biosynthesis pathway (R/Bioconductor package) ^38^.
